# An Evil Which Must Be Ended: Night Duty Torments for Patients

**Published:** 1919-01-18

**Authors:** Paul Pry


### THE EDITOR'S LETTER-BOX—(*continued*).

### An Evil Which Must be Ended: Night Duty Torments for Patients.

*To the Editor of* THE HOSPITAL.

SIR,—I thank "Ancon," but does it not seem of sad significance that the only instance so far given of an hospital where day duties are not mixed up with those of the night refers to an hospital not in England? I hope the last word has not been said, however. It would be too bad if in England—where we think we lead in the intelligent care of our sick and the intelligent training of our nurses—none could tell of an hospital, *and* training school, where washings are not done, nor beds made, in what practically is the middle of the night. I myself have known a Colonial hospital where the duties of day and night nurses were from six to six, and all washings, bedmaking, and breakfasts the exclusive duties of the day nurses—nothing but the absolutely necessary wants of patients (for treatment and comfort) the business of the night nurses, nor any duties but those of nursing at all. In this way, which from my own experience worked very well, one night nurse could look well after twenty-four patients on the one floor, to which in day-time three nurses would be allotted.

There were none of those two—or three—off duty hours, either for day or night workers, but one whole day, or night, for each nurse weekly, to ensure which sufficient nurses were always on relief duties. That was, however, an hospital of about seventy beds only.

I sampled another Colonial hospital (this time as patient), considered a first-class training-school for nurses and in every way up to date, where the worst night-duty faults distinguishing the "Old Country" were in full swing. Lights were lowered at 8 P.M., after mag. sulph. had been freely given round to about a third of the patients each night. At nine the night nurses relieved the day ones and stayed to talk quite loudly for about half an hour—topics generally plays and matinées. Towards eleven one "houseman" usually came, who "walked and talked" with the nurse in the ward for at least another half-hour on subjects that had nothing to do with nursing —which all could hear. This young doctor, too, would always wake up any patient of his who chanced to be asleep to ask how she was, which proceeding of course woke up patients who were not his. This over, the nurse had lavatories and bathroom to clean, which made noise enough to keep awake patients already wakened; then she made up afresh any bed vacated too late the day before for the attentions of the day probationer. Sometimes, if a patient were known to be going out next morning, that patient would be requested about 2 A.M. to finish the night on a couch so that the night nurse could make the bed up afresh for the next patient, clean the locker out, etc. And at four o'clock, or earlier, if other things were done, the nurse began the washing, bedmaking, and other attentions required by the eight patients allotted to her—all surgical operations not allowed, or able, to do anything for themselves. At six she gave breakfast to the whole ward of some twenty beds. What sleep could the patients get with all that disturbance going on, not to mention the evening dosings of mag. sulph.? PAUL PRY.

[We are grieved to find that no letters have been sent by matrons, sisters, or nurses who must be aware of the existence of night-duty torments for patients in British hospitals. Must the existing evils be published with the names of the hospitals concerned before the responsible officials speak or will take effective action? The College ought to act decisively and quickly in this matter. No properly conducted hospital must suffer these evils to continue. Wake up, committee men and women.—ED., THE HOSPITAL.]

### "Discharged Officers and their Outlook."

*To the Editor of* THE HOSPITAL.

